# The QuitIT Coping Skills Game for Promoting Tobacco Cessation Among Smokers Diagnosed With Cancer: Pilot Randomized Controlled Trial

**DOI:** 10.2196/10071

**Published:** 2019-01-10

**Authors:** Paul Krebs, Jack Burkhalter, Jeffrey Fiske, Herbert Snow, Elizabeth Schofield, Michelle Iocolano, Sarah Borderud, Jamie S Ostroff

**Affiliations:** 1 Department of Population Health New York University School of Medicine New York, NY United States; 2 Department of Psychiatry and Behavioral Sciences Memorial Sloan-Kettering Cancer Center New York, NY United States; 3 Muzzy Lane Software Newburyport, MA United States

**Keywords:** tobacco, cancer, mHealth, app, mobile phone

## Abstract

**Background:**

Although smoking cessation apps have become popular, few have been tested in randomized clinical trials or undergone formative evaluation with target users.

**Objective:**

We developed a cessation app targeting tobacco-dependent cancer patients. Game design and behavioral rehearsal principles were incorporated to help smokers identify, model, and practice coping strategies to avoid relapse to smoking. In this randomized pilot trial, we examined feasibility (recruitment and retention rates), acceptability (patient satisfaction), quitting self-confidence, and other cessation-related indices to guide the development of a larger trial.

**Methods:**

We randomized 42 English-speaking cancer patients scheduled for surgical treatment to either the Standard Care (SC; telecounseling and cessation pharmacotherapies) or the experimental QuitIT study arm (SC and QuitIT game). Gameplay parameters were captured in-game; satisfaction with the game was assessed at 1-month follow-up. We report study screening, exclusion, and refusal reasons; compare refusal and attrition by key demographic and clinical variables; and report tobacco-related outcomes.

**Results:**

Follow-up data were collected from 65% (13/20) patients in the QuitIT and 61% (11/18) in SC arms. Study enrollees were 71% (27/38) females, 92% (35/38) white people, and 95% (36/38) non-Hispanic people. Most had either lung (12/38, 32%) or gastrointestinal (9/38, 24%) cancer. Those dropping out were less likely than completers to have used a tablet (*P*<.01) and have played the game at all (*P*=.02) and more likely to be older (*P*=.05). Of 20 patients in the QuitIT arm, 40% (8/20) played the game (system data). There were no differences between those who played and did not play by demographic, clinical, technology use, and tobacco-related variables. Users completed an average of 2.5 (SD 4.0) episodes out of 10. A nonsignificant trend was found for increased confidence to quit in the QuitIT arm (*d*=0.25, 95% CI −0.56 to 1.06), and more participants were abstinent in the QuitIT group than in the SC arm (4/13, 30%, vs 2/11, 18%). Satisfaction with gameplay was largely positive, with most respondents enjoying use, relating to the characters, and endorsing that gameplay helped them cope with actual smoking urges.

**Conclusions:**

Recruitment and retention difficulties suggest that the perihospitalization period may be a less than ideal time for delivering a smoking cessation app intervention. Framing of the app as a “game” may have decreased receptivity as participants may have been preoccupied with hospitalization demands and illness concerns. Less tablet experience and older age were associated with participant dropout. Although satisfaction with the gameplay was high, 60% (12/20) of QuitIT participants did not play the game. Paying more attention to patient engagement, changing the intervention delivery period, providing additional reward and support for use, and improving cessation app training may bolster feasibility for a larger trial.

**Trial Registration:**

ClinicalTrials.gov NCT01915836; https://clinicaltrials.gov/ct2/show/NCT01915836 (Archived by WebCite at http://www.webcitation.org/73vGsjG0Y)

## Introduction

Smartphones and tablet computers are approaching universal use and have opened up new possibilities for the delivery of smoking cessation intervention apps [1,2]. As of 2015, 441 English-language smoking cessation apps were available in app stores [3]. Significant possibilities exist for the development of evidence-based smoking cessation apps targeted to meeting the needs of specific populations of smokers, including young adults, persons with chronic health conditions, etc.

While hundreds of smoking cessation apps are available, few have been subjected to clinical trials or formative evaluation with target users [3,4]. A review of the scientific literature through 2015 found only 3 cessation apps that had been tested in a randomized trial design [3]. This lack of research is concerning because 66% of users of mobile health (mHealth) apps report logging in at least once a day, and 55% users spend at least 11 minutes interacting with them [5]. In addition, most (97%) existing tobacco-related apps have not incorporated principles of effective behavior change [6]. The largest category of smoking-related apps are “calculators,” which track money saved and other quitting metrics. Studies have demonstrated, however, that this simple approach does not help smokers quit [4,7,8].

Cessation apps also often fail to take advantage of features to improve user engagement [9]. In a national survey of health app developers, about half of respondents (46%) had stopped using some health apps, primarily due to high data entry burden, loss of interest, and hidden costs [5]. Gamification or “the application of game design principles in order to change behaviors in nongame situations” [10] offers potential to engage mHealth users and promote behavior change [11-14]. Gamification of smoking cessation interventions has led to higher reported levels of engagement in a comparison of mobile apps that featured education and progress tracking [15]. For instance, the Super Smoky game, which focused on youth smokers, was found to increase motivation to quit smoking [16]. However, depth of gameplay was still limited and consisted of having users turn their avatar away from cigarettes and smoking opportunities and did not contain comprehensive information on quitting.

Our research group has developed a smoking cessation intervention based on game design principles and behavioral rehearsal therapy to help smokers identify, model, and practice coping strategies to avoid relapse to smoking [17-19]. Our development process included expert focus groups, prototyping with game developers, and think-aloud testing with a sample of 20 smokers with a history of cancer [18]. We developed the game for use with hospitalized smokers due to our observation of high relapse following hospital discharge. Effective smoking cessation and relapse prevention interventions require participant engagement in a range of complex challenges and strategies such as identifying tobacco use triggers, engaging in alternative coping behaviors, seeking social support from family and friends about tobacco use, modifying one’s internal dialogue, and dealing with inevitable slips to prevent relapse [20]. Translating these evidence-based strategies into a game required an immersive app in which users can learn and practice these techniques in a realistic context. Through repeated exposure to conditioned cues to smoke, (eg, socializing with friends who smoke), such a game environment may be able to help smokers virtually practice and master coping skills and build crucial self-efficacy for managing smoking urges. Our premise is that an intervention that combines virtually presented smoking cues with engaging narrative and personally relevant coping skills practice may help smokers overcome barriers to quitting and maintaining tobacco abstinence. The goal of the project was to develop a cessation treatment app using an immersive laptop or tablet-based game environment to help smokers cope with smoking urges and prevent smoking lapses. After developing the game through a formative evaluation process, we conducted a randomized pilot study to examine the feasibility of conducting a subsequent clinical trial (NCT01915836), its acceptability, and preliminary data regarding the game’s effects on tobacco cessation coping and use outcomes.

## Methods

### Participants and Procedures

Memorial Sloan-Kettering Cancer Center (MSKCC) patients who reported being current smokers at their initial medical appointments were advised to quit by their attending physician and referred to the MSKCC Tobacco Treatment Program. Research staff contacted potentially eligible patients via telephone to describe study procedures and screen for additional eligibility criteria. Eligible participants were English-speaking patients with a recent (within the past 6 months) cancer diagnosis or mass suspicious of cancer, those scheduled for surgery to remove a localized tumor, those who reported smoking cigarettes within the past 30 days, and those who had sufficient sensory acuity and manual dexterity to use a computer game. Those with a distant metastatic disease, major psychiatric illness, cognitive impairment, and inability to comply with study procedures or provide informed consent were excluded from the study. The research assistant (RA) met participants in the hospital a day or two following their surgery to complete informed consent and baseline assessments and to provide training on the use of the intervention.

### Randomization or Design

Participants were stratified by age (<65, ≥65 years) and randomly assigned with a 1:1 ratio to two study arms. We stratified by age to control for the potential relationship between age and prior mobile devices experience. Participants completed a baseline survey at enrollment and a follow-up survey via phone or mail 1 month following hospital discharge.

### Intervention Conditions

#### Standard Care

Participants were offered at no cost 4 telephone or bedside counseling sessions and our in-house print cessation educational materials [21]. Trained oncology nurses certified as tobacco treatment specialists who follow evidence-based behavioral and pharmacological best practices conducted the counseling sessions. The initial session focused on motivation building, choosing a quit date, if applicable, reviewing the print educational materials, and providing information about and arranging the provision of cessation pharmacotherapies. The second counseling session focused on coping with smoking urges and preventing smoking relapse. The third and fourth counseling sessions focused on relapse prevention or recycling to a repeat quit attempt for those who had resumed smoking. At the end of each session, the tobacco treatment specialists completed a checklist outlining the goals of each session to track patient adherence and treatment fidelity.

#### Standard Care + Smoking Cues Coping Skills Game (QuitIT)

Patients assigned to the QuitIT condition were offered Standard Care (SC) in addition to having the QuitIT game installed on an iPad device. The RA trained participants during their hospital stay on how to use the game. Training sessions included a verbal overview of the game, its rules, and its objectives. Then, participants were asked to watch a brief tutorial video, play a practice game episode, and afterwards, the RA evaluated patients’ comprehension of gameplay. Patients were encouraged to play 3-4 game episodes per week for a 1-month period posthospitalization. Participants were loaned an iPad for 1 month and instructed to contact the RA if they encountered technical difficulties.

The intervention used gaming techniques to exemplify key behavioral strategies based on the social cognitive theory [18]. The game was conceptualized in a narrative structure meant to engage users in each of 10 episodes featuring different characters across 9 situations, all depicting common smoking-related triggers. These included getting ready in the morning, coming home from work, driving in a car, having a frustrating phone conversation, and being offered a cigarette while drinking with friends. The goal of each episode was for users to guide the character through a series of tempting situations and thoughts without resorting to smoking. Users clicked on response choices to guide the character and direct the story line. The screen presented an “urge to smoke” meter, which helped prompt users to monitor and engage the character in appropriate strategies. If a player did not assist the character, the character would slip and smoke, presenting the user with feedback and the opportunity to try again. After users successfully negotiated an episode, the game moved on to the next one. Users received points and badges for avoiding smoking and completing episodes. Multimedia Appendix 1 shows 3 screenshots from the intervention. Screenshot 1 shows the main game screen; here one can see progress across the 9 scenarios and tutorial as well as the badges that users have earned (on the right column). New scenarios get unlocked when one is completed, and users can replay previous scenarios again to earn more points and badges. Badges represent the various coping strategies users employ in the scenarios, with the idea that they should diversify their coping strategies to earn different badges. Screenshot 2 shows a prototypical challenge scenario, in this case, getting the character Ann out of the apartment without smoking. Across the top of the screen are meters showing that Ann’s triggers in this scene are nicotine withdrawal and pain. In addition, we see her smoking urge meter. Users click on the cards at the bottom to choose her next action. The cards represent various coping strategies, including cognitive self-talk, relaxation strategies, and using cessation medications, as well as a distractor card that contains a nonhelpful choice that would increase her smoking urge. In screenshot 3, the challenge is for Liz to not smoke during a night out. Here the challenges at the top are wanting to celebrate and feel social; she has chosen a behavioral strategy, switching to drinking ginger ale to stay in control of the situation and avoid smoking. To reinforce coping strategies outside of gameplay, we provided participants with a set of real-life “coping cards,” which resembled playing cards and outlined the primary coping strategies in the game and featured scenes from the game.

### Measures

#### Demographics and Medical Status

At baseline, participants reported demographics including age, sex, ethnicity, education, occupation, comorbid medical conditions, and smoking and quitting history. Medical charts were reviewed, and data on cancer diagnosis and treatment were extracted.

#### Tobacco Use

At baseline, tobacco use and quitting history were assessed with standard measures adapted from the National Adult Tobacco Survey [22]. One month after study entry (1 month), smoking abstinence (“have you smoked combustible cigarettes, even a puff, in the last 7 days?”), relapse (“Have you smoked cigarettes, even a puff, since you were discharged from the hospital?”), quitting attempts (“Have you tried to quit smoking since you entered the study?”), and use of cessation medications and other interventions were assessed via self-report. Abstinence was biochemically verified with salivary cotinine assays. Saliva samples were collected and analyzed for cotinine concentrations using gas chromatography, consistent with standardized methods. Active, passive, and no smoking exposure were defined as cotinine concentrations of ≥31.5 ng/mL, 0.5-31.4 ng/mL and <0.5 ng/mL, respectively [23]. For participants reporting follow-up use of nicotine replacement therapies or electronic cigarettes, breath samples were conducted in person to test for levels of expired carbon monoxide (CO). Tobacco abstinence was confirmed by <10 ppm CO in the expired air.

#### Tobacco-Related Variables

At both baseline and 1-month follow-up, the following measures were assessed: (1) the Fagerstrom Test of Nicotine Dependence, a 6-item self-report scale with a summary score between 0 (very low dependence) and 10 (heavy dependence) [24], was used to assess dependence; (2) the 10-item Questionnaire of Smoking Urges-brief form [25] measured the intensity of smoking urges; and (3) the Confidence Questionnaire assessed situational self-efficacy in being able to resist urges to smoke across 16 everyday situations [26].

#### Gameplay

The game database tracked the number of log-ins, unique sessions, length of play, and episodes completed.

#### Satisfaction

Questions regarding participants’ experience, satisfaction, and perceived game helpfulness were implemented in-game after 30 minutes of gameplay and at 1-month follow-up for participants to evaluate their experiences using the QuitIT game. We used 10 items developed by our research team to evaluate general experiences with the game and perceived helpfulness to improve the subjective experience of the game for future iterations.

### Analysis

The primary goal of this pilot study was to examine feasibility (recruitment and retention rates), acceptability (patient satisfaction), and cessation-related trends associated with the intervention to guide the development of a larger trial. We, therefore, detail screening, exclusion, and refusal reasons and compare refusal and attrition by key demographic and clinical variables. For tobacco-related outcomes, we report means and SDs at baseline and 1-month follow-up. Between-group differences were not analyzed on tobacco-related outcomes as the pilot was not powered to detect statistically significant differences. Analyses were conducted using SAS version 9.3 (SAS Institute, Inc) and the MBESS package in R (version 3.3.3) for CIs of effect sizes.

## Results

### Participants

**Figure 1:**
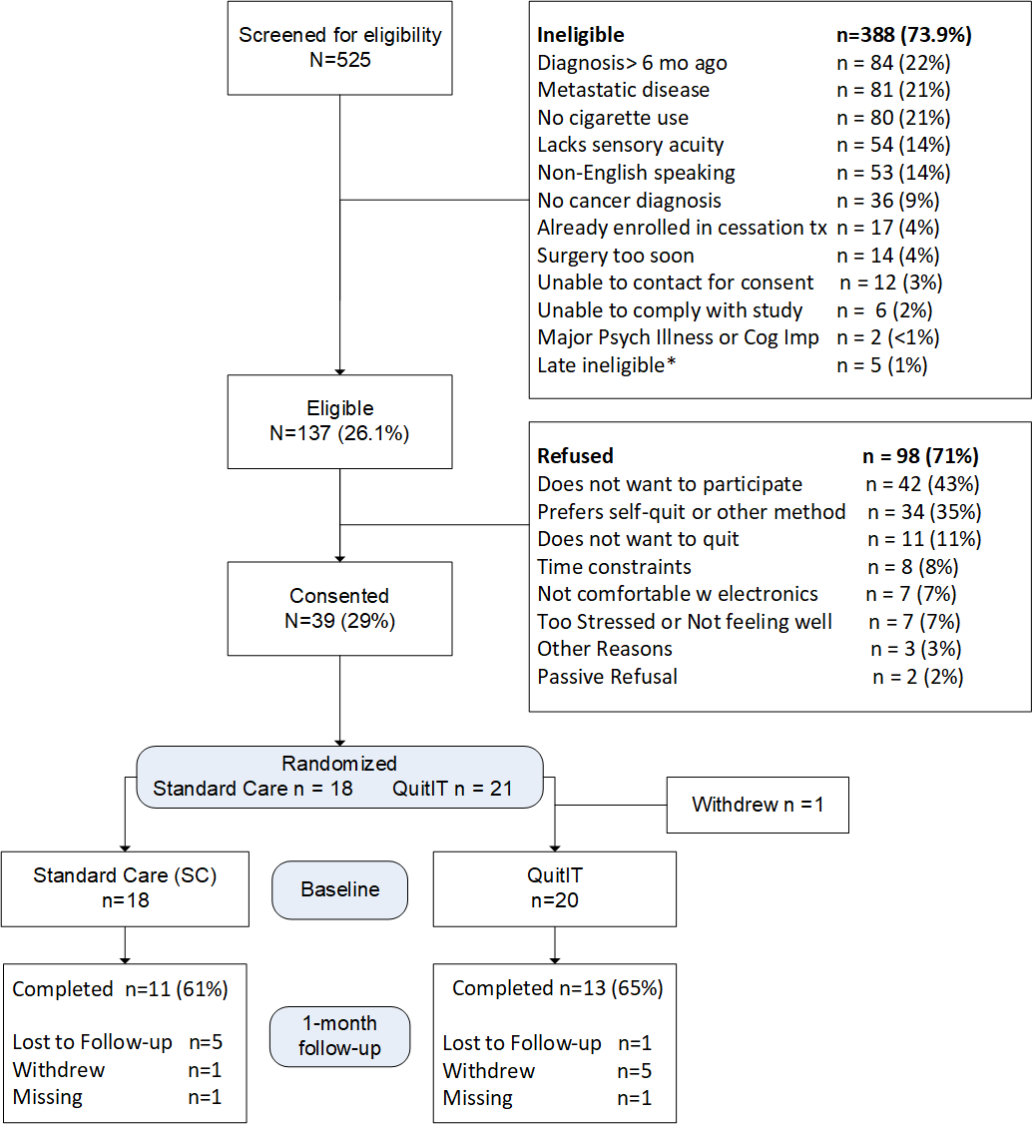
Study flow. Asterisk indicates that five participants were deemed ineligible after randomization due to a change in prognosis; their data were not analyzed. Cessation tx: cessation treatment; cog imp: cognitive impairment; psych illness: Diagnostic and Statistical Manual of Mental Disorders, Fifth Edition psychological diagnosis.

A total of 525 patients were screened for participation. Of those, 388 were determined to be ineligible, primarily because of having been diagnosed >6 months ago, not having smoked in the past 30 days, and having metastatic disease. Of 137 eligible patients, 71% (98/137) refused, reporting that they were not interested in the study (n=42), preferred to quit on their own (n=34), did not want to quit at all (n=11). A total of 38 patients were randomized. At 1-month follow-up, data were collected from 13 patients in the QuitIT arm and 11 in the SC arm (see Figure 1 for full study flow details).

Table 1 shows that 40% (15/38) of enrolled participants were between the age of 50 and 59 years, 71% (27/38) were females, 92% (35/38) were white people, and 95% (36/38) were non-Hispanic people. The most common cancer diagnoses were lung (12/38, 32%) and gastrointestinal (9/38, 24%) cancers. Most patients were diagnosed with disease stages I (14/38, 37%) or II (7/38, 18%).

### Feasibility and Use Metrics

Refusal and attrition rates were examined to assess the feasibility of conducting a future intervention trial. Those who enrolled versus refused were more likely to be female (*P*=.003) but did not differ by other demographic or clinical characteristics (see Table 1). Across both QuitIT and SC arms, a total of 24 participants completed the 1-month follow-up. As shown in Table 2, those who less frequently used a tablet computer at baseline were more likely to drop out (*P*<.01), less likely to have used the game at all (*P*=.02), and more likely to be older (*P*=.05). Of 20 in the QuitIT arm, 40% (8/20) played the game (as determined by system data). No statistically significant differences in demographic, clinical, technology use, and tobacco-related variables were found between those who played and did not play (Multimedia Appendix 2). Users completed an average of 2.5 (SD 4.0) episodes, with a range of 0-10 episodes completed.

### Tobacco-Related Outcomes

At 1-month assessment, data were available from 24 participants. A trend was found for the primary tobacco-related outcome, increased situational self-efficacy (confidence to quit), in the QuitIT arm (*d*=0.25, 95% CI –0.56 to 1.06). In addition, the QuitIT participants reported higher intention to stay quit (*d*=1.03, 95% CI 0.14-1.89; Table 3). Confirmed abstinence was higher in the QuitIT arm, with 30% (4/13) of the sample reporting abstinence versus 18% (2/11) in the SC arm. Nicotine replacement therapy and other cessation medications were used by a minority of participants in each arm with 5/11 (46%) using them in the SC and 4/13 (31%) in the QuitIT arm.

### Satisfaction and Participant Feedback

At 1-month follow-up, 8 participants who used the game completed survey items related to satisfaction and helpfulness (Table 4). Most respondents thought the game kept their attention and 63% (5/8) thought it was fun to use. Of them, 88% (7/8) said they could relate to the characters and 63% (5/8) indicated that they got interested in their stories. All participants said they learned at least something about coping with smoking urges. A little more than half (63%, 5/8) thought playing helped them cope with urges to smoke; 75% (6/8) would apply what they learned in real life and learned at least a moderate amount from the coping cards and 75% (6/8) thought the game was the right length.

**Table 1:** Comparison of participant characteristics by enrollment status.

Characteristic			All (n=136), n (%)		Enrolled (n=38), n (%)		Refused (n=98), n (%)		*P* value^a^
**Age (years)**								.50	
	<40	7 (5.2)		3 (7.9)		4 (4.1)			
	40-49	15 (11.0)		4 (10.5)		11 (11.2)			
	50-59	52 (38.2)		15 (39.5)		37 (37.8)			
	60-69	45 (33.1)		13 (34.2)		32 (32.7)			
	70+	17 (12.5)		3 (7.9)		14 (14.3)			
**Sex**								.003	
	Female	69 (50.7)		27 (71.1)		42 (42.9)			
	Male	67 (49.3)		11 (29.0)		56 (57.1)			
**Race**								.18	
	White	114 (83.8)		35 (92.1)		79 (80.6)			
	Black	7 (5.2)		1 (2.6)		6 (6.1)			
	Asian	4 (2.9)		2 (5.3)		2 (2.0)			
	Other	1 (0.7)		0 (0)		1 (1.0)			
	Refused	10 (7.4)		0 (0)		10 (10.2)			
**Ethnicity**								.54	
	Non-Hispanic	131 (96.3)		36 (94.7)		95 (96.9)			
	Hispanic	5 (3.7)		2 (5.3)		3 (3.1)			
**Cancer site**								.08	
	Gastrointestinal	40 (29.4)		9 (23.7)		31 (31.6)			
	Lung	32 (23.5)		12 (31.6)		20 (20.4)			
	Urologic	18 (13.2)		2 (5.3)		16 (16.3)			
	Colorectal	14 (10.3)		5 (13.2)		9 (9.2)			
	Gynecologic	11 (8.1)		6 (15.8)		5 (5.1)			
	Other	21 (15.4)		4 (10.5)		17 (17.4)			
**Cancer stage**								.72	
	0	7 (5.2)		3 (7.9)		4 (4.1)			
	I	54 (39.7)		14 (36.8)		40 (40.8)			
	II	27 (19.9)		7 (18.4)		20 (20.4)			
	III	26 (19.1)		6 (15.8)		20 (20.4)			
	IV	7 (5.2)		4 (10.5)		3 (3.1)			
	Missing (or 88 or 99)	15 (11.0)		4 (10.5)		11 (11.2)			

^a^*P* value is based on *t* test for age, Mantel-Haenszel chi-square for clinical stage, and Pearson’s chi-square for all other variables.

**Table 2:** Attrition by participant characteristics (n=38).

Characteristic		All (n=38)	Completers (n=24)	Lost to follow-up (n=14)	*P* value	
**Sex, n (%)**						.48
	Female	27 (71)	18 (67)	9 (33)		
	Male	11 (29)	6 (55)	5 (46)		
**Race, n (%)**						.34
	White	35 (92)	21 (60)	14 (40)		
	Asian	2 (5)	2 (100)	0 (0)		
	Black	1 (3)	1 (100)	0 (0)		
**Marital status, n (%)**						.28
	Married	21 (55)	11 (52)	10 (48)		
	Divorced or widowed	11 (29)	8 (73)	3 (27)		
	Single	6 (16)	5 (83)	1 (17)		
**Education, n (%)**						.42
	≤High school	9 (24)	4 (44)	5 (56)		
	Some college	12 (32)	9 (75)	3 (25)		
	College grad	17 (45)	11 (65)	6 (35)		
**Employment, n (%)**						.20
	Employed	14 (37)	9 (64)	5 (36)		
	Retired	13 (34)	6 (46)	7 (54)		
	Unemployed or on leave	11 (29)	9 (82)	2 (18)		
**Income, n (%)**						.66
	<US $10k	4 (11)	4 (100)	0 (0)		
	US $10k-$30k	4 (11)	2 (50)	2 (50)		
	US $30k-$70k	8 (21)	4 (50)	4 (50)		
	>US $70k	20 (53)	13 (65)	7 (35)		
	Missing	2 (5)	1 (50)	1 (50)		
**Baseline tablet use, n (%)**						<.01
	Never or rarely or monthly	14 (37)	4 (29)	10 (71)		
	Occasionally or more	24 (63)	20 (83)	4 (17)		
**Baseline gameplay history, n (%)**						.85
	Never or rarely or monthly	6 (16)	4 (67)	2 (33)		
	Occasionally or more	32 (84)	20 (63)	12 (38)		
**Smoking since diagnosis, n (%)**						.97
	Maintained or increased	8 (21)	5 (63)	3 (38)		
	Decreased	30 (79)	19 (63)	11 (37)		
**Quit attempts of >24 hours in past year, n (%)**						.33
	No	9 (24)	4 (44)	5 (56)		
	Yes, once	9 (24)	7 (78)	2 (22)		
	Yes, more than once	20 (53)	13 (65)	7 (35)		
**Cancer site, n (%)**						.62
	Lung	12 (32)	7 (58)	5 (42)		
	Gastrointestinal	9 (24)	4 (44)	5 (56)		
	Gynecologic	6 (16)	5 (83)	1 (17)		
	Colorectal	5 (13)	4 (80)	1 (20)		
	Urologic	2 (5)	1 (50)	1 (50)		
	Other	4 (11)	3 (75)	1 (25)		
**Clinical stage, n (%)**						.46
	0	3 (8)	1 (33)	2 (66.7)		
	I	14 (37)	8 (57)	6 (42.9)		
	II	7 (18)	5 (71)	2 (28.6)		
	III	6 (16)	3 (50)	3 (50.0)		
	IV	4 (11)	3 (75)	1 (25.0)		
	Missing (or 88 or 99)	4 (11)	4 (100)	0 (0)		
**Arm, n (%)**						.80
	Standard Care	18 (47)	11 (61)	7 (39)		
	Quit It	20 (53)	13 (65)	7 (35)		
**Gameplay (n=20), n (%)**						.02
	Any	8 (21)	8 (100)	0 (0)		
	None	30 (79)	16 (53)	14 (47)		
Age (current; years), mean (SD)		57.11 (9.6)	54.79 (9.1)	61.07 (9.5)	.05	
Baseline Fagerstrom score^a^, mean (SD)		3.68 (2.2)	3.41 (2.1)	4.17 (2.5)	.35	
Years smoking (n=40), mean (SD)		36.7 (12.6)	34.43 (14.1)	40.50 (8.8)	.16	
Baseline cigarettes per day (n=41), mean (SD)		12.34 (14.7)	11.10 (7.1)	14.46 (22.8)	.50	
Baseline intention to abstain for 30 days (n=40), mean (SD)		2.73 (2.1)	3.04 (2.3)	2.15 (1.8)	.23	
**Baseline coping strategies, mean (SD)**						.57
	Number used (of 13)	8.47 (3.8)	8.75 (4.2)	8.00 (3.2)		
**Baseline situational self-efficacy, mean (SD)**						.76
	Mean (of 16 items), range 0-100	57.05 (22.6)	56.20 (24.2)	58.52 (20.2)		

^a^0-2: Very low; 3-4: Low; 5: Medium; 6-7: High; 8-10: Very high or heavy.

**Table 3:** Tobacco-related outcomes at 1-month follow-up.

Characteristic	Standard care (n=11), mean (SD)	QuitIT (n=13), mean (SD)	Effect size *d* (95% CI)
Situational self-efficacy (range 0-100)	80.01 (19.3)	84.43 (15.9)	0.25 (−0.56 to 1.06)
Days abstinent postdischarge	25.64 (11.8)	23.85 (23.3)	−0.09 (−0.90 to 0.71)
Length of admission (days)	8.64 (5.9)	6.69 (5.0)	0.36 (−0.45 to 1.17)
Intend to abstain for next 30 days (range 1-5)	1.55 (0.9)	3.75 (2.8)	1.03 (0.14 to 1.89)
Importance of quitting (range 1-10)	10 (0)	9.46 (1.9)	−0.38 (−1.18 to 0.44)

**Table 4:** Satisfaction with the game at 1-month follow-up (n=8).

Item and response		Value, n (%)
**The game kept my attention.**		
	Strongly agree or agree	6 (75)
	Neither agree nor disagree	0 (0)
	Disagree or strongly disagree	2 (25)
**The game has been fun to use.**		
	Strongly agree or agree	5 (63)
	Neither agree nor disagree	2 (25)
	Disagree or strongly disagree	1 (13)
**I could relate to the characters as they dealt with smoking temptations.**		
	Strongly agree or agree	7 (88)
	Neither agree nor disagree	0 (0)
	Disagree or strongly disagree	1 (13)
**I got interested in the characters’ stories.**		
	Strongly agree	5 (63)
	Neither agree nor disagree	1 (13)
	Disagree or strongly disagree	2 (25)
**How much did you learn from the game about ways to help you cope with smoking urges?**		
	I learned a lot	4 (50)
	I learned a moderate amount	2 (25)
	I learned a little bit	2 (25)
	I didn’t learn much at all	0 (0)
**Playing the game helped me cope with urges to smoke.**		
	Strongly agree	5 (63)
	Neither agree nor disagree	0 (0)
	Disagree or strongly disagree	3 (38)
**How likely are you to apply what you learned in the game to real like smoking temptations?**		
	Extremely likely or likely	4 (50)
	Likely	2 (25)
	Neither likely nor unlikely	2 (25)
	Unlikely or extremely unlikely	0 (0)
**How would you rate your experience with this game session?**		
	Extremely satisfied	2 (25)
	Satisfied	3 (38)
	Neither satisfied nor dissatisfied	2 (25)
	Dissatisfied or extremely dissatisfied	1 (13)
**In terms of length, the game sessions:**		
	Took too long	2 (25)
	Were just about right	6 (75)
	Were too short	0 (0)
**How useful were the game cards in helping you cope with smoking urges?**		
	Extremely helpful	2 (25)
	Very helpful	2 (25)
	Moderately helpful	1 (13)
	A little bit helpful	1 (13)
	Not at all helpful	2 (25)
**In the past month, how many days did you look at the deck of QuitIT cards?**		
	0	3 (38)
	1-4	2 (25)
	5-19	0 (0)
	20+	2 (25)
	Missing	1 (13)

## Discussion

This study presents findings from a pilot randomized controlled trial evaluating acceptability, use, and preliminary outcomes from an interactive tablet-based game to promote abstinence from tobacco use following cancer-related hospitalization. Results should be interpreted with regard to the feasibility of pursing a larger powered trial. The following criteria were assessed as indicators of whether to pursue follow-up work: recruitment of the target sample size in the allotted timeframe, acceptance rate of at least 50%, retention of at least 80%, at least small effect sizes (*d*=0.2) on primary outcomes, minimal adverse events, and patients’ reports of interest in and acceptability of the intervention [27]. In light of these criteria, our goal of 190 patients was not met primarily due to insufficient number of eligible patients (N=137) despite screening all Tobacco Treatment Program-referred patients for 2 years. Among those eligible, 71% (98/137) declined participation citing lack of interest in the study (42/98, 43%) or preferring to quit on their own (34/98, 35%) as the most common reasons. The study retention was 61% (11/18) and 65% (13/20) in the SC and QuitIT conditions, respectively, which is lower than the 80% feasibility criterion.

Recruiting study participants in the context of recent cancer diagnosis and treatment is complicated by patient anxiety, disruptions in daily patterns due to multiple medical appointments, and worry about treatment outcomes. Nonetheless, the recruitment rate is markedly lower than observed in other cessation studies we have conducted with patients recently diagnosed with cancer. For example, we found a 30% recruitment rate in a previous trial testing a handheld computer that guided smoking reduction with presurgical, tobacco-dependent, cancer patients [28]. Other studies [7,15,16,29] that have examined cessation apps were not clinical trials or used Web-based volunteer recruitment, precluding ascertainment of recruitment rates from a specified cohort of patients. Nevertheless, the low recruitment rate warrants consideration for future app studies. It is possible that describing the app as a “game” may have appeared as inappropriately frivolous in the context of a cancer diagnosis and surgery, contributing to the low rate of study participation. Using a more serious term such as “mobile app or guide for smoking cessation,” “video simulation,” or “games for behavior change” may be more appropriate to the cancer context. We primarily used a telephone recruitment approach, which presents difficulties for explaining such a novel intervention to potential participants. Recruiting participants in person, or having an interactive ad on the hospital website, during which the intervention could be demonstrated may increase interest and willingness to participate. In addition, 35% (34/98) noted they wanted to quit on their own and refused all cessation services, suggesting optimistic bias and, perhaps, low awareness of the effectiveness of cessation interventions [30]. Further work, including qualitative interviews with this population, may assist in framing app-based interventions for future trials.

Although the study was not powered to detect a difference in quit rates, 6 people had confirmed smoking abstinence. Despite this, more participants in the QuitIT group were abstinent than the SC arm. In addition, while not statistically significant, there was a trend for increased confidence in quitting for the QuitIT arm (*d*=0.25), as well as for intention to abstain (*d*=1.03). These moderate effects suggest some potential promise for the intervention and meet the specified criteria for feasibility [27]. Given that only 8 of 21 people in the QuitIT arm actually played the game and only for an average of 2.5 sessions, it is likely that these treatment effects might be more pronounced with greater adherence to gameplay or app use. This suggests that the game likely has potential, but that increased attention should be paid to addressing barriers for use. Gameplay during perihospitalization and recovery may be particularly challenging due to the healing process and presence of physical symptoms adversely affecting energy and quality of life. Introducing the game at another time and a longer intervention period with more frequent prompts to play the game may ameliorate these challenges.

In terms of app use and acceptability, satisfaction data from the 1-month follow-up indicate that users found that the game kept their attention and was fun to use, but that it could be improved in terms of relating to the characters and helping cope with urges. In addition, there were no study-related adverse events. It may be necessary to provide an additional reward for use and to support use with counselor check-ins, or monetary incentives [31], as well as improved training, as noted previously. We found that prior tablet use and younger age were found to be significant predictors of app use; this is not surprising given that younger age has been associated with the health app use [5]. Persons who already owned a tablet would be more familiar and likely to integrate it into their daily activities, using it for reading, email, internet, etc, whereas, persons who received it only for the study would not otherwise interact with the tablet (study tablets were locked down for other uses). Older persons with greater income and education are just as likely as younger persons to use smartphones and tablets [32], but also less likely to be smokers [33]. Our sample of cancer patients skews toward older persons, who would likely require increased coaching about tablet use as well as technical support. One training session was likely insufficient to help nonusers become comfortable with using a tablet. Furthermore, allowing participants to use the tablet for other activities would likely increase instances of app use.

This pilot clinical trial is, nevertheless, an important advance in establishing an evidence base for health-related apps, and tobacco cessation apps, in particular. The great majority of tobacco cessation-related apps are of poor quality [4]. In an analysis of apps being guided by behavior change theory, Choi et al found that only 10.3% (18/175) apps examined used 3 key theoretical domains [34]. Of apps that have been developed by smoking behavior change experts, *QuitSTART* from Smokefree.gov and the Truth campaign’s *This is Quitting* app have not yet been evaluated in a clinical trial. *SmartQuit*, an app based on the Acceptance and Commitment therapy, was found to have superior engagement compared with the *QuitSTART* app [7,35]. Greater evidence for apps is necessary to assess this treatment modality and promote greater use. Only 20% of health app users have had a doctor recommend an app [5]. This is not surprising as a recent survey of 264 health care providers found that although most (203/264, 76.9%) believed that apps had potential to change smoking behavior, fewer (112/264, 42.4%) believed that the currently available apps were useful in treatment [9]. Our findings suggest that better patient engagement and greater participant training are essential for conducting trials of apps in clinical populations and that greater adherence is needed to properly assess intervention effects. Next steps should involve another round of formative qualitative interviews with potential users to identify how to improve descriptions of the app to improve recruitment, increase adherence to game use, and meet expectations for help to improve skills for coping with smoking urges.

## Appendix

Multimedia Appendix 1 Screenshots from game.

Multimedia Appendix 2 Baseline characteristic differences by actual gameplay (n=20 in QuitIT arm).

Multimedia Appendix 3 CONSORT‐EHEALTH checklist (V 1.6.1).

## Abbreviations

CO: carbon monoxide
MSKCC: Memorial Sloan-Kettering Cancer Center
RA: research assistant
SC: standard care
