# Supplementary material for: The QuitIT Coping Skills Game for Promoting Tobacco Cessation Among Smokers Diagnosed With Cancer: Pilot Randomized Controlled Trial
Source: JMIR Mhealth Uhealth. 2019 Jan 10;7(1):e10071. doi: 10.2196/10071 (PMC6329892; doi:10.2196/10071)

Multimedia Appendix 1

Screenshot 1: Main game screen with progress through scenarios

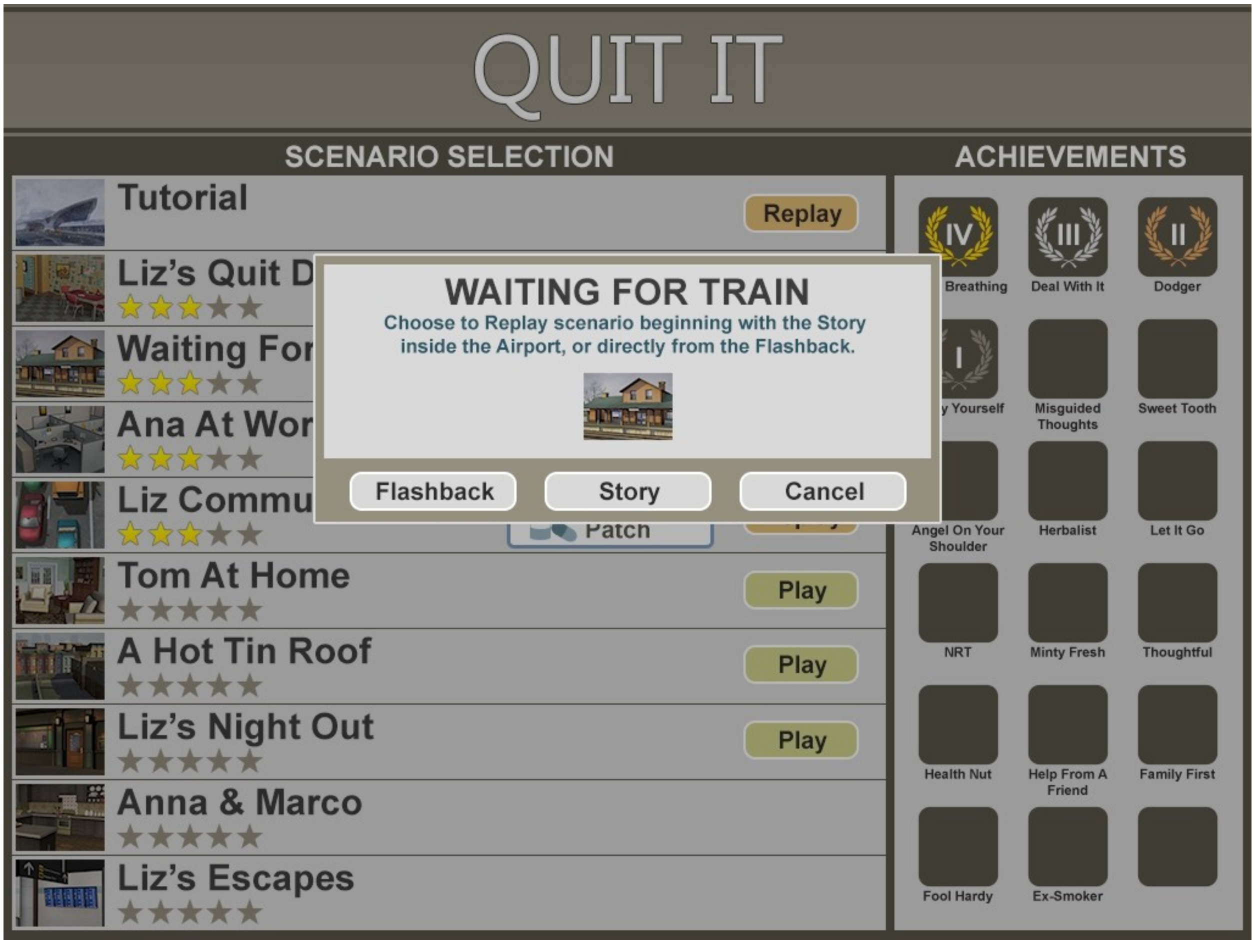

Screenshot 2: Ann at home choosing coping strategies

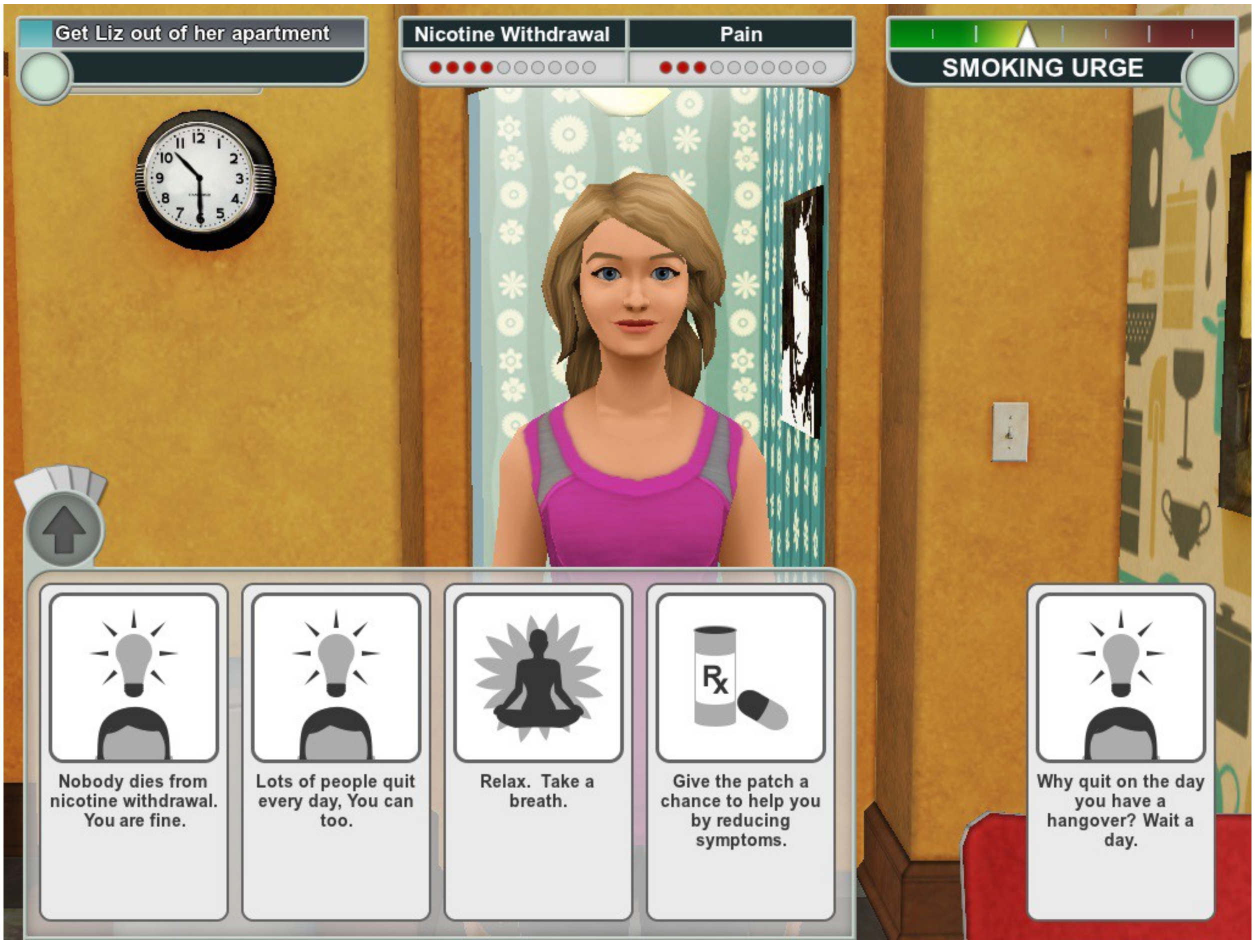

Screenshot 3: Liz at a bar making a coping decision

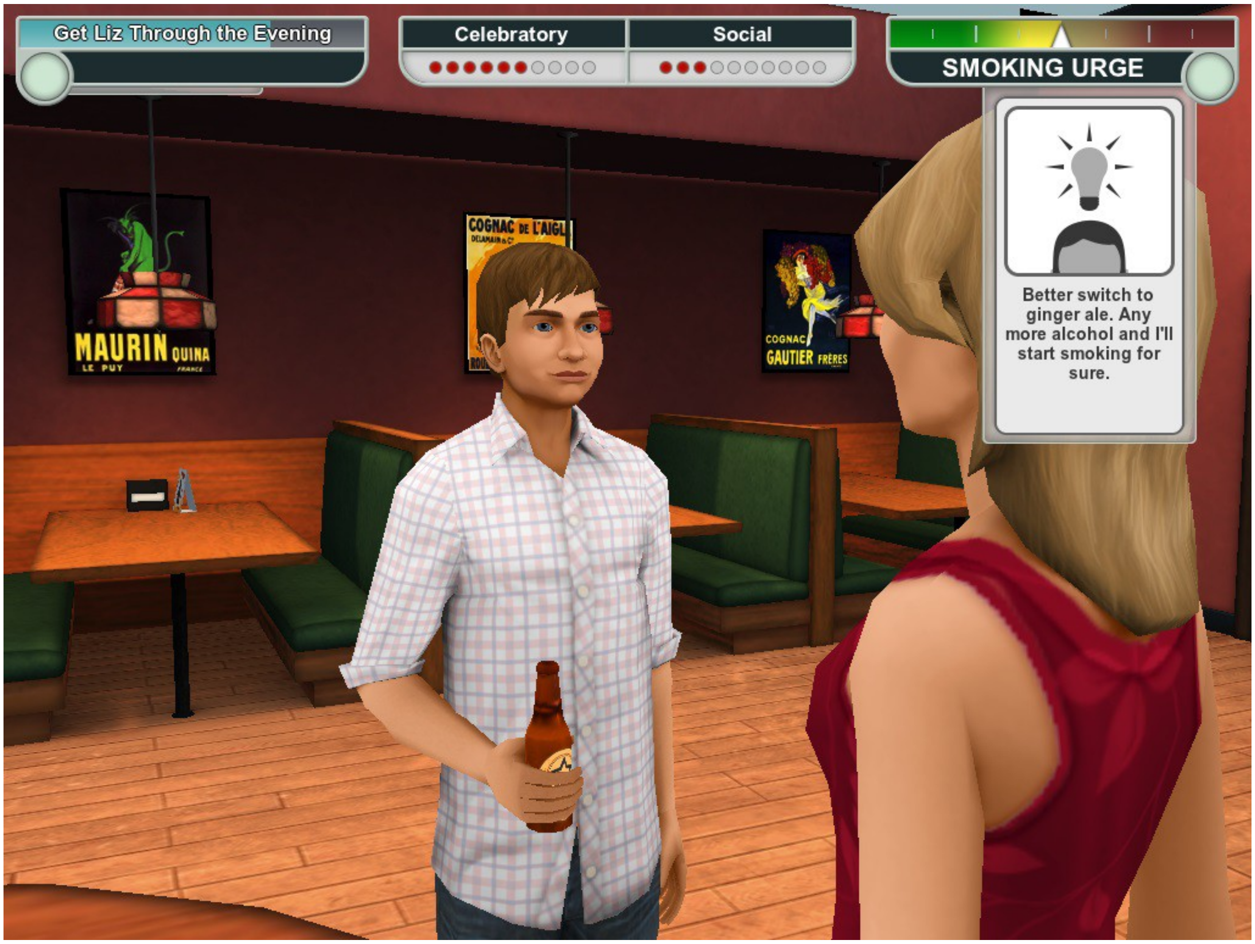

Supplement: Multimedia Appendix 1 [file mhealth_v7i1e10071_app1.pdf]
