# Supplementary material for: The QuitIT Coping Skills Game for Promoting Tobacco Cessation Among Smokers Diagnosed With Cancer: Pilot Randomized Controlled Trial
Source: JMIR Mhealth Uhealth. 2019 Jan 10;7(1):e10071. doi: 10.2196/10071 (PMC6329892; doi:10.2196/10071)
Supplement: Multimedia Appendix 2 [file mhealth_v7i1e10071_app2.pdf]

Multimedia Appendix 2.

Baseline Characteristic Differences by Actual Gameplay (n=20 in QuitIt Arm)

**Characteristic**	**Category**	**All (n=20)**	**No Play (n=12)**	**Play[1] (n=8)**	**P value[2]**
Sex, n (%)					>.99
	Female	13	7 (54)	6 (46)	
	Male	7	5 (71)	2 (29)	
Race, n (%)					0.73
	White	16	10 (63)	6 (38)	
	Black	3	2 (67)	1 (33)	
	Other	1	0 (0)	1 (100)	
Marital Status, n (%)					0.41
	Married	10	7 (70)	3 (30)	
	Single	4	3 (75)	1 (25)	
	Divorced or Widowed	6	2 (33)	4 (67)	
Education, n (%)					>.99
	HS or less	3	2 (67)	1 (33)	
	Some college	4	2 (50)	2 (50)	
	College grad	13	8 (62)	5 (38)	
Employment, n (%)					0.30
	Employed	9	4 (44)	5 (56)	
	Retired	7	6 (86)	1 (14)	
	Unemployed/ on leave	4	2 (50)	2 (50)	
Income, n (%)					0.22
	<$10k	2	2 (100)	0 (0)	
	$10k - $30k	2	1 (50)	1 (50)	
	$30k - $70k	5	4 (80)	1 (20)	
	>$70k	10	5 (50)	5 (50)	
	Missing	1	0 (0)	1 (100)	
Baseline Tablet Use , n (%)					0.20
	Never/Rarely/ monthly	9	7 (78)	2 (22)	
	Occasionally or more	11	5 (45)	6 (55)	
Baseline Game Play, n (%)					>.99
	Never/Rarely/ monthly	2	1 (50)	1 (50)	
	Occasionally or more	18	11 (61)	7 (39)	
Smoking since diagnosis, n (%)					0.62
	Maintained or increased	4	3 (75)	1 (25)	
	Decreased	16	9 (56)	7 (44)	
Quit attempts of >24 hours in past year, n (%)					0.31
	No	2	2 (100)	0 (0)	
	Yes, once	5	4 (80)	1 (20)	
	Yes, more than once	13	6 (46)	7 (54)	
Cancer Site, n (%)					0.58
	Colon/ Rectum/ Prostate/ Testes	3	2 (67)	1 (33)	

	Panc/ Stom/ Kidney/ Bladder	6	5 (83)	1 (17)	
	Lung or Bronchus	4	2 (50)	2 (50)	
	Other	7	3 (43)	4 (57)	
	Missing	2	1 (50)	1 (50)	
Clinical Stage, n (%)					0.06
	0	6	2 (33)	4 (67)	
	I	1	1 (100)	0 (0)	
	II	1	1 (100)	0 (0)	
	III	3	3 (100)	0 (0)	
	IV	7	4 (57)	3 (43)	
	Missing (or 88 or 99)	13	7 (54)	6 (46)	
Age, mean (SD)		56.4 (11.0)	58.4 (12.3)	53.4 (8.6)	0.33
Years smoking, mean (SD)		35.6 (15.9)	36.3 (16.0)	34.5 (16.8)	0.82
Cigarettes per day, mean (SD)		14.8 (18.9)	16.3 (24.0)	12.6 (7.4)	0.69
Age started smoking, mean (SD)		21.4 (9.3)	19.8 (8.1)	23.8 (11.0)	0.37
Days abstained from smoking (n=17), mean (SD)		21.3 (25.8)	27.7 (30.3)	14.2 (19.1)	0.30
Intention to Abstain for 30 days, mean (SD)		2.7 (2.1)	2.6 (2.0)	2.9 (2.3)	0.77
Coping strategies (number used of 13), mean (SD)		8.1 (3.7)	7.3 (4.0)	9.3 (2.9)	0.26
Situational Self-Efficacy (of 16 items) range 0-100, mean (SD)		20.3 (28.9)	15.8 (29.1)	26.9 (29.2)	0.42

[1]Includes one participant for whom we do not have game play data but know to have completed at least some game play.

[2]P values are based on t-tests for continuous variables (e.g., age and age started), Mantel-Haenszel Chi-square for education, income, and and stage, and Fisher's exact test for remaining categorical variables.
